# Visible light‐responsive azo‐based smart materials: Design, performance, and applications in energy storage

**DOI:** 10.1002/smo.20240058

**Published:** 2024-12-04

**Authors:** Shurui Lv, Yuang Zhang, Wentao Wang, Shufen Zhang, Bingtao Tang

**Affiliations:** ^1^ State Key Laboratory of Fine Chemicals Frontier Science Center for Smart Materials Dalian University of Technology Dalian China; ^2^ School of Materials Science and Engineering Zhejiang Sci‐Tech University Hangzhou China

**Keywords:** energy storage materials, photochemistry

## Abstract

Azobenzene and its derivatives are the most extensively investigated and applied molecular photoswitches, which can undergo reversible transformation between *trans* and *cis* isomers upon irradiation with light at specific wavelengths. Through structural geometry transformation, the property alterations can be integrated into smart materials to meet diverse application requirements. Most azo‐based photoswitches require UV light for activation. However, complete activation within the visible or even near‐infrared light range could offer several benefits for photoswitch applications, including improved biocompatibility, better light penetration, and enhanced solar light utilization efficiency. This review presents an overview of the development of visible‐light responsive azo‐based materials, covering molecular design strategies and their applications in energy storage. Recent efforts aimed at enhancing the performance of azo‐based energy storage materials are highlighted. According to the different strategies for improving energy storage properties, these materials are categorized as those that directly increase isomerization energy and those that introduce phase transition energy. Furthermore, we discuss the challenges and opportunities in this field with a view to inspire further exploration.

## Graphical abstract

Azobenzene and its derivatives are the most extensively investigated and applied molecular photoswitches, which can undergo reversible transformation between *trans* and *cis* isomers upon irradiation with light at specific wavelengths. Through structural geometry transformation, the property alterations can be integrated into smart materials to meet diverse application requirements. Most azo‐based photoswitches require UV light for activation. However, complete activation within the visible or even near‐infrared light range could offer several benefits for photoswitch applications, including improved biocompatibility, better light penetration, and enhanced solar light utilization efficiency. This review presents an overview of the development of visible‐light responsive azo‐based materials, covering molecular design strategies and their applications in energy storage. Recent efforts aimed at enhancing the performance of azo‐based energy storage materials are highlighted. According to the different strategies for improving energy storage properties, these materials are categorized as those that directly increase isomerization energy and those that introduce phase transition energy. Furthermore, we discuss the challenges and opportunities in this field with a view to inspiring further exploration.

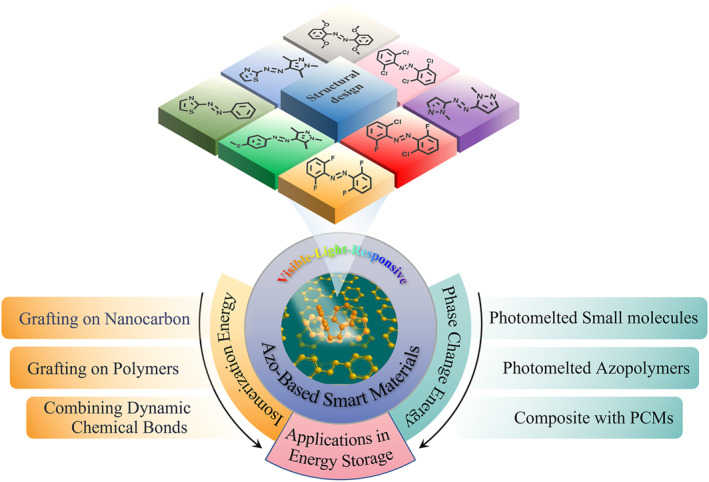

## 1 INTRODUCTION

Smart‐responsive materials, a novel class of functional materials, can detect a variety of external stimuli (e.g., light, electricity, magnetism, heat, humidity, pH) and generate corresponding adaptive responses. Among these stimuli, optical stimulation is characterized by its multidimensional tunability, enabling remote control with high spatiotemporal precision and intrinsic cleanliness. All these features have propelled photoresponsive materials to the forefront of global research, establishing them as leading contenders in the current landscape of science and technology.^[1]^

**FIGURE 1:**
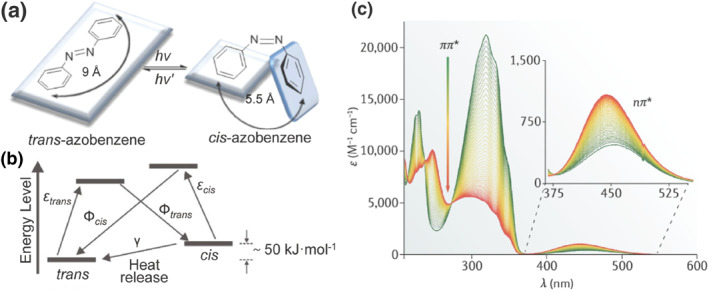
Comprehensive analysis of azobenzene photoisomerization: (a) Mechanistic pathway, Reproduced with permission: Copyright 2012, Merino and Ribagorda, published by Beilstein‐Institut.^[3]^ (b) Isomer energy levels, Reproduced with permission: copyright 2019, John Wiley and Sons.^[4]^ (c) Absorption spectra. Reproduced with permission: Copyright 2019, Springer Nature.^[5]^

The discovery of azo compounds, characterized by a conjugated aromatic ring system bridged by an azo bond, dates back to the mid‐19th century. Nevertheless, it was not until 1937 that G.S. Hartley unveiled the phenomenon of photoisomerization in these compounds.^[2]^ Azo compounds exhibit *trans* (*E*) and *cis* (*Z*) isomerism, showing distinct spectral, geometric, and thermodynamic differences. The *E* isomer is a (quasi) planar rod with a size of 9 Å and a dipole moment of 0.5 D. The *Z* isomer is curved with a size of 5.5 Å and a dipole moment of 3.0 D. In contrast, the *Z* configuration has a significantly lower length, but the spatial volume of the molecule increases substantially and the molecular polarity increases (Figure 1a).^[3]^ In addition, the *E* isomer is more stable due to its lower ground state energy, while the *Z* isomer is thermodynamically unstable.

Particularly, the reversible transition between the two isomers can be achieved through light irradiation, which lays the foundation for their use as molecular switches in smart light‐responsive materials. Azo compounds exhibit notable photochemical stability, outstanding fatigue resistance, prompt photic responses, and absorption spectra align well with conventional light sources. These attributes, combined with their straightforward synthesis and the ability to adjust properties through structural modifications, establish them as leading light‐responsive materials in research.

Under specific illumination conditions, an azo compound reaches a photo‐stationary state (PSS). At this state, the relative abundances of the *E* and *Z* isomers in the system achieve an equilibrium. As shown in Figure 1b, the composition of the PSS is unique to each system, dictated by the quantum yields for the two processes (Φ_*trans*_ and Φ_*cis*_) and the thermal relaxation rate constant (γ). Among them, the quantum yield determines the effectiveness of molecular response to light, while the thermal relaxation rate constant affects the relative thermal stability of the *cis* isomer. Moreover, the photoisomerization of azo compounds is also affected by factors such as irradiation intensity, wavelength, and temperature.^[4]^ The absorption spectral signature of the azo compound features n→π* and π→π* transition leaps due to a lone pair of electrons on the nitrogen atom. During *trans*‐to‐*cis* isomerization process, spectral alterations primarily include a slight shift and enhancement in the n→π* band, along with a significant blueshift and decrease in the π→π* band (Figure 1c). The symmetry‐forbidden n→π* transitions (S_0_→S_1_, HOMO→LUMO) correspond to the less intense band in the visible region, whereas the symmetry‐allowed π→π* transitions (S_0_→S_2_, HOMO‐1→LUMO) correspond to a stronger intense band in the UV range.^[5]^ Thus, it becomes clear why UV light is essential for triggering the isomer switching, given the significant absorbance intensity difference between the two isomers in this wavelength range.

However, it is important to note that UV light not only has a relatively low penetration depth but also can be harmful to cells and tissues. Similarly, it poses a threat to the integrity of organic and polymeric substances. As a result, an increasing number of researchers have been focusing on designing visible light‐responsive azo molecules to enhance their applicability. Strategies for achieving visible light responsiveness are primarily categorized into two types: direct and indirect. The direct strategy focuses on modulating the absorption spectrum through molecular design to achieve visible light responsiveness while the indirect methods employ sensitizers like upconversion nanoparticles to facilitate energy or electron transfer, thereby enabling activation by visible light. Previous review articles have systematically summarized these strategies.[^6^, ^7^, ^8^]

Azo‐based materials have been known for a long time, but their use as energy storage materials is a recent development. Their stable and reversible photoisomerization make them highly significant for energy storage research and applications.^[9]^ Decades ago, photoisomerization reactions were identified as a potential approach for solar energy utilization.^[10]^ The *E* isomer of an azo photoswitch can absorb light energy and convert to the *Z* isomer as well as storing energy. The energy difference between the *Z* and *E* isomers represents the stored isomeric energy in the material. Therefore, the proportion of *Z* isomerization at the photo‐stationary state has a significant effect on the energy storage density. When the metastable *Z* isomer reverts to the stable *E* isomer, the stored energy is released as heat.^[11]^ This provides a scientific foundation for their use in energy storage applications.

**FIGURE 2:**
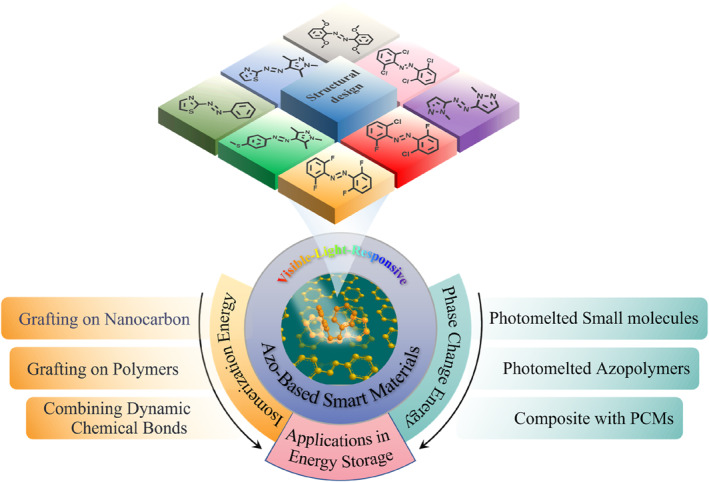
Overview of enhancing methods for azo‐based smart materials in energy storage. (The molecules shown in the figure are reported azo photoswitches responsive to visible light[^12^, ^13^, ^14^, ^15^, ^16^, ^17^, ^18^, ^19^]).

In this review, we first discuss the structures of azo molecules designed to respond to visible light through direct strategies, which summarized the major findings of previous research. Then, we explore the applications of azo‐based photoresponsive materials in energy storage, highlighting examples of visible light response. On this basis, the review classified the efforts to enhance the energy storage performance of materials. These efforts are categorized into two parts: directly improving the isomerization energy of the system and introducing phase change energy. It is hoped that, through clever molecular and structural design, the requirements for energy storage in applications can be met (Figure 2). Finally, we summarized the challenges encountered and provided an outlook for future developments of azo‐based smart materials in the field of energy storage, aiming to provide reference for relevant researchers.

## 2 STRUCTURAL ENGINEERING

**FIGURE 3:**
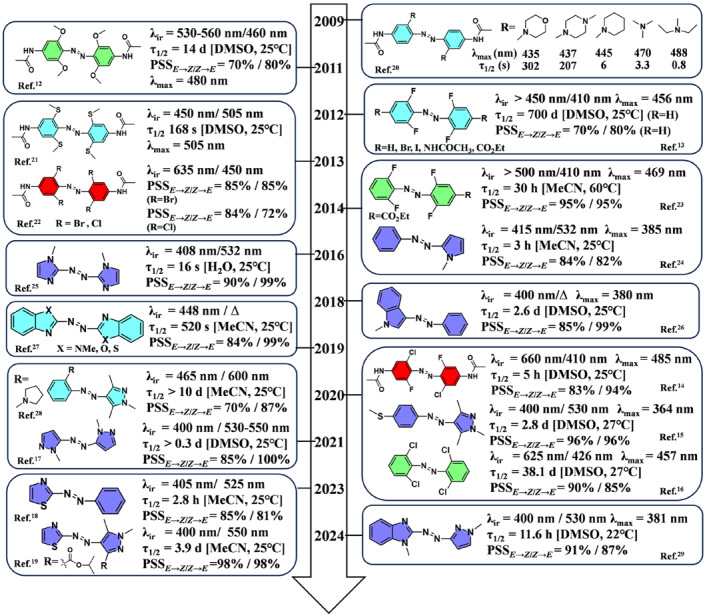
Molecular structures and characteristics of visible‐light‐responsive azo photoswitches.[^12^, ^13^, ^14^, ^15^, ^16^, ^17^, ^18^, ^19^, ^20^, ^21^, ^22^, ^23^, ^24^, ^25^, ^26^, ^27^, ^28^, ^29^]

The structure of azo compounds can be precisely designed at the molecular level to adjust their maximum absorption peaks, half‐life, and other properties, which forms the basis for improved functionality. Among the various visible light‐responsive azo molecules, we focus on some classic structures that are widely used in practice as well as heteroaryl azo compounds that have drawn significant attention in recent years. Classic structures, such as *ortho*‐substituted azo compounds, are valued for their advantageous half‐life, high isomerization yields, and efficient synthesis. These attributes significantly contribute to the advancement of visible‐light‐driven azo‐based smart materials. Expanding the scope of exploration, recent studies have identified azoheteroarenes as a promising frontier due to their unique attributes in advanced light‐responsive materials. Figure 3 chronologically aggregates the molecular designs in a concise format.

### 2.1 *Ortho*‐substituted azobenzene derivatives

The most straightforward approach for designing visible‐light switches on a molecular scale is to extend the π system in order to reduce the HOMO‐LUMO gap and hence lead to redshift its absorption.^[20]^ Amino‐azobenzene compounds, featuring electron‐donating groups (EDGs) *ortho* or *para* to the N=N bond, are classic examples of visible‐light azo switches. Woolley et al. used a 4,4′‐diacetamidoazobenzene core, and added EDG amino substituents at the 2,2′‐positions, which result in redshifting the absorption maximum to 370–513 nm.^[20]^ The extent of redshift correlates with the electron‐donating potency of the substituents. However, this approach compromises the *Z*‐isomer's lifetime (*τ*_1/2_ = 0.5–209 s at 20°C), which hinders its practical applicability.

**FIGURE 4:**
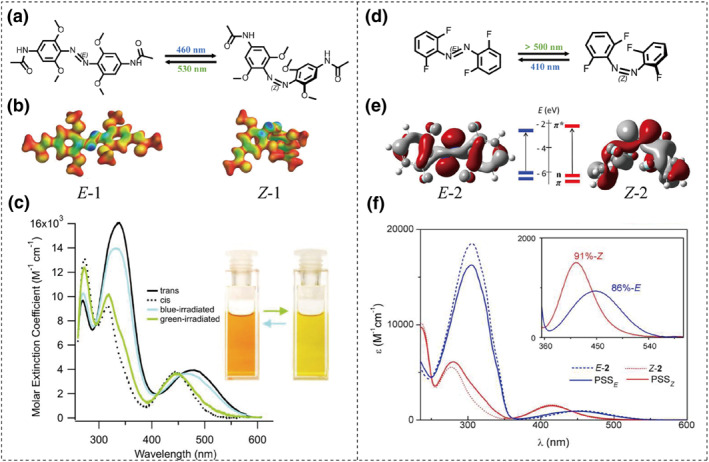
(a) Photoisomerization of *ortho*‐tetra‐methoxy azobenzene **1**. (b) Calculated structures of *E*‐1 and *Z*‐1. The absolute value of the HOMO of each isomer is mapped onto the bond density surface (blue is the maximum value of the HOMO). (c) UV–vis absorption spectra of *E*/*Z* isomers of **1** in DMSO. Reproduced with permission: Copyright 2011, American Chemical Society.^[12]^ (d) Photoisomerization of *ortho*‐tetra‐fluoro‐azobenzene **2**. (e) Energetic diagram of the π, n, and π* orbitals of **2**. (f) UV–vis absorption spectra of *E*/*Z* isomers of **2** in acetonitrile. Reproduced with permission: Copyright 2012, American Chemical Society.^[13]^

To slow down the rate of thermal *cis* to *trans* relaxation and avoid photobleaching, the Woolley group developed a classical method by substituting the 2,2′,6,6′ position of 4,4′‐diacetamidoazobenzene with the weaker electron donor methoxy group (**1**), as shown in Figure 4a.^[12]^ Figure 4b shows the calculated structures of the *E*‐ and *Z*‐ (**1**). For the trans isomer, the electron‐rich oxygen atoms of the methoxy groups increase the repulsion with the HOMO orbital, thereby raising its energy. This results in a significant redshift of the n→π* absorption peak compared to the *Z* isomer, which ensures effective separation (Figure 4c). As a result, bidirectional isomerization can be simply achieved by employing green light (530–560 nm) for the *trans*‐to‐*cis* conversion and blue light (450–460 nm) for the *cis*‐to‐*trans* return. Notably, this modification extends the *Z*‐isomer's half‐life in phosphate buffer to approximately 2.4 days, which is suitable for most biological applications. This also paves the way for azobenzene photoswitches to achieve visible light‐driven bistable reversible isomerization.

Subsequently, the group reported that replacement of oxygen atoms with sulfur at the ortho positions improved its application in a biological context.^[21]^ Nevertheless, the overlap of the absorption bands of the *cis* and *trans* isomers can have a negative impact on the isomerization yield.

Stefan Hecht's group reported on a novel class of visible‐light‐responsive azo compounds known as *ortho*‐fluoro‐azobenzene (**2**), as shown in Figure 4b.^[13]^ Incorporating fluorine, a strong electron‐withdrawing atom, can reduce the electronic repulsion across the N=N bond and stabilizes the n‐orbital in the *Z*‐isomer (Figure 4d). Consequently, the energy of the *Z*‐isomer n→π* transition increases and blueshifts, which result in a clear 42 nm separation between the n→π* bands of *Z* and *E* isomers (Figure 4f). This enables the use of visible light for the reversible isomerization with high quantum yields. Moreover, fluorine atom's compact size helps to maintain the planarity of the *E*‐isomer, similar to that of conventional azobenzene.

It is worth noting that the *cis* isomer exhibits remarkably high thermal stability, owing to a significant decrease in the n‐orbital's energy, which results in a half‐life of up to 700 days in DMSO at 25°C. Subsequent research by this group demonstrated that the properties of fluorine‐substituted azobenzene switches are tunable. By altering substituent position, number, and type of substituents, they could affect excitation wavelength, photoconversion, and *Z*‐isomer longevity.^[23]^

Subsequently, some researchers have explored properties differences to replace the fluorine atom with other halogen atoms, such as the chlorine or the bromine.[^14^, ^22^, ^30^] These photoswitches undergo a *trans*‐to‐*cis* transition upon red light stimulation, which offers advantages in certain areas, particularly in biology and medicine.

*Ortho*‐fluoroazobenzenes have garnered significant attention because of their capability to achieve high‐efficiency bidirectional isomerization under visible light combined with the exceptional thermal stability of their *cis*‐isomer. Introducing functional groups at the ortho positions is a classical strategy to enable visible light responsiveness in azo compounds. Ortho substituents, particularly methoxy groups and halogens, are known to slow down the thermal back‐isomerization process. This approach has been widely utilized in many fields to improve the photomodulation function of systems, including liquid crystal materials,[^31^, ^32^, ^33^, ^34^, ^35^, ^36^, ^37^] supramolecular assemblies,[^38^, ^39^, ^40^] hydrogels,[^41^, ^42^, ^43^] biofunctional molecules,[^44^, ^45^, ^46^, ^47^] and porous frame material.[^48^, ^49^, ^50^]

### 2.2 Azoheteroarenes

Azoheteroarenes have begun to attract attention recently.[^5^, ^51^, ^52^] Replacing one or both aryls with a heteroaryl allows for a broader exploration of modulating the photochromic properties of azo molecules due to the wide variety of heteroaryls.

**FIGURE 5:**
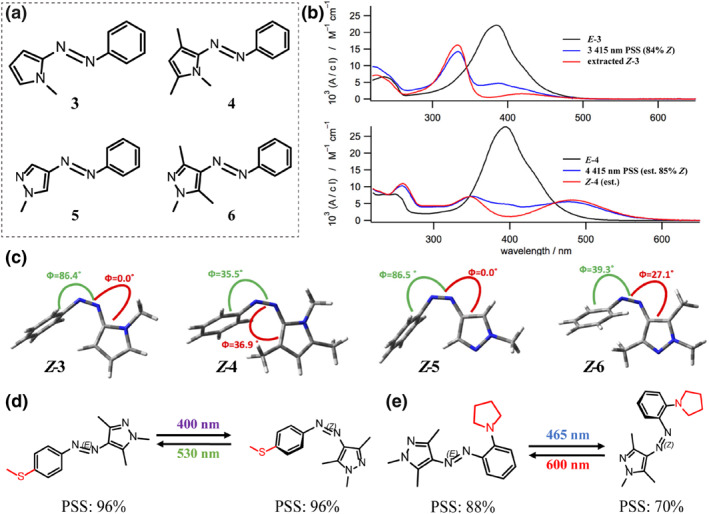
(a) Structures of the heteroarylazoes **3–6**. (b) UV‐vis spectra of azoheteroarenes **3** and **4** in acetonitrile. (c) Calculated conformations for *Z* isomers (B3LYP/6‐31G(d,p)), with dihedral angles annotated. All values, except Φ_NNhet_ for *Z*‐5, are given as 180‐Φ. Reproduced with permission: Copyright 2014, American Chemical Society.^[24]^ (d) Photoisomerization of arylazopyrazoles **7** and **8**.

Fuchter and colleagues reported a series of N‐substituted heteroarylazoes (Figure 5a).^[24]^ As shown in Figure 5b, the pyrroles in **3** and **4** exhibit strong p‐π conjugation effects, which enhance their π‐donating ability to the azo function and result in a significant red shift in the π–π* absorption peak. The redshift enables bidirectional isomerism under visible light stimulation (415/532 nm). The *cis* isomers of **3** and **4** have significantly different half‐lives, 21 h and 21 s, respectively. They conducted systematic theoretical calculations to elucidate the relationship between the molecular structure and isomeric properties of these azoheteroarenes (Figure 5c). In **3**'s *cis* isomer, the pyrrole ring is coplanar with the azo bond, and the benzene ring is perpendicular to the plane of the pyrrole ring‐azo bond, forming a symmetric T‐configuration. In contrast, azoheteroarenes **4**'s *cis* isomer has a highly asymmetric twisted configuration due to the steric hindrance of the methyl group, which enhances the n‐π* absorption peak and favors photoisomerization. Bis‐methylation of azoheteroarenes **4** introduced spatial repulsion, which increased the *cis*‐isomer's energy. Meanwhile, dispersive forces reduce the energy of the transition state, thereby lowering thermal isomerization barriers and speeding up the *cis*‐isomer's recovery.

Significantly, azopyrazole *Z*‐**5** and *Z*‐**6** also demonstrate the relationship between their geometrical configuration and photoisomeric properties as described previously (Figure 5c). Azopyrazole **5** exhibits a T‐configuration with an extensive half‐life of 1000 days at room temperature (25°C), whereas azopyrazole **6**, with a twisted configuration, achieves near‐quantitative bidirectional isomerization but has a reduced half‐life of 10 days. These findings have significantly sparked researchers' interest in this area of study. Subsequently, the same research group extended the half‐life of azopyrazole **6** up to an impressive 46 years by introducing a methoxy or halogen element, such as fluorine, in the neighboring position.^[53]^

Unlike traditional azo compounds, well‐established synthetic methods such as diazo coupling, the Mills reaction, and oxidation of aromatic amines often fail to construct various heteroaryl azo molecules. Surprisingly, the synthesis of compounds **5** and **6** involves a new method for in situ formation of heterocycles, which opens up new avenues for the efficient synthesis of heteroaryl azo compounds. However, the *trans*‐to‐*cis* isomerization processes of these molecules still require UV excitation.

Scientists have enhanced azoheteroarenes **6**'s photosensitivity to visible light. Samanta et al. investigated *ortho*‐ and *para*‐thiomethyl substituted arylazopyrazoles (AAPs), showcasing efficient isomerization under visible light, with *para*‐substitution enabling near‐quantitative photoisomerization (96%) bidirectionally (Figure 5d).^[15]^ Ravoo's team optimized photochromic characteristics of AAPs by introducing pyrrolidine (pyr) and piperidine (pip) substituents to the benzene ring. These modifications induced an intense visible absorption band in the spectrum of the *E* isomer.^[28]^ Consequently, the optimized compound could be efficiently actuated using blue light (*λ* = 465 nm) for *E*‐to‐*Z* isomerization and red light (*λ* = 600 nm) for the reverse *Z*‐to‐*E* transition (Figure 5e). Adjacent pyr or pip groups notably extend the compounds' half‐life over *ortho*‐substituted variants. The enhanced half‐life originates from weak non‐covalent interactions between *o*‐substituents and the methylated pyrazole ring.

**FIGURE 6:**
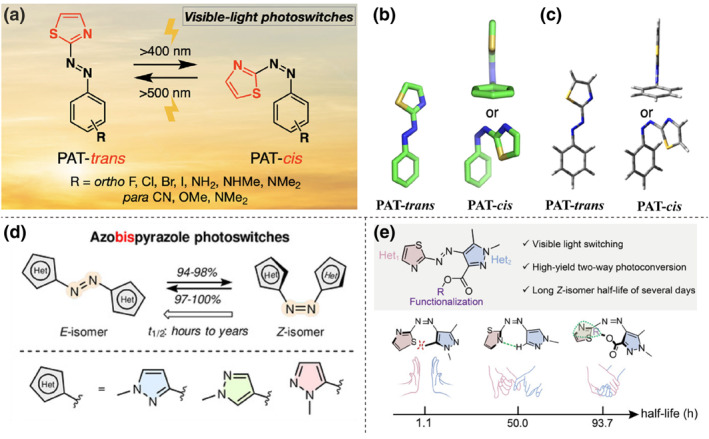
(a) Phenylazothiazoles as visible‐light photoswitches. (b) Single‐crystal X‐ray structures and (c) geometry‐optimized calculated conformations of the *E* and *Z* isomers of PAT (green = C; blue = N; gold = S; red = Cl) are shown. Hydrogens are omitted for clarity. Reproduced with permission: Copyright 2014, American Chemical Society.^[18]^ (d) Molecular design and reversible isomerization of azobispyrazoles. Reproduced with permission: copyright 2021, John Wiley and Sons.^[17]^ (e) Thiazolylazopyrazoles as nonsymmetric bis‐heteroaryl azo switches. Reproduced with permission: copyright 2023, John Wiley and Sons.^[19]^

Beyond substituent modifications, researchers have harnessed novel heterocycles to enable visible light responsiveness in azo compounds. König's team synthesized a series of phenylazoindoles that can isomerize under 400 nm light, a property not shared by arylazopyrroles.^[26]^ This unique behavior is attributed to the extended conjugation system in the benzopyrrole moiety, which causes a significant redshift in the π‐π* transition. Tamaoki et al. unveiled a suite of phenylazothiazole (PAT) compounds, as shown in Figure 6a. These compounds harness the electron‐donating sulfur in the thiazole ring, which notably narrows the π‐π* energy gap, thereby enabling efficient isomerization under visible light through enhanced absorption capabilities.^[18]^ Remarkably, similar to arylazopyrazoles, the *Z* isomers of these five‐membered azoheteroarenes can assume a T‐conformation, characterized by a heteroarene‐azo unit positioned nearly orthogonal to the phenyl group, thus enhancing their half‐life (Figure 6b,c). The stability of this T‐shaped conformation in arylazo thiazoles largely hinges on the delocalization of the sulfur atom's lone pair electrons into the benzene ring's antibonding π* orbital. When the thiazole S atoms are oriented toward the benzene ring, the p‐π* conjugation is optimized, which results in stabilizing the T conformation. Conversely, the introduction of an electron‐donating substituent at the para position of the benzene ring weakens this beneficial interaction, leading to a reduced half‐life. The development of various heteroaryl rings provides a vast exploration space for tuning the properties of azo‐based photoswitches.

Recent advancements highlight bis‐heteroaryl azo compounds (Het‐N=N‐Het) as a versatile platform for tailoring electronic properties in photoswitches. Fuchter's group crafted an azobis (2‐imidazole) switch, utilizing the pyrrole N's proximity to the azo bond for redshifted absorption, enabling efficient *E*/*Z* interconversion under visible light, aided by the *Z*‐isomer's twisted conformation.^[25]^ Similarly, Beves et al. developed symmetric azobenzazole switches with benzimidazole, benzoxazole, or benzothiazole groups, showcasing visible‐light responsiveness through absorption maxima in the visible spectrum.^[27]^ Li et al. innovated with azobispyrazole switches, featuring bilateral pyrazole units (Figure 6d).^[17]^ By varying the azo‐pyrazole linkage, they modulated the *Z*‐isomer's half‐life from hours to years. The pyrrole‐type N's electron‐donating effect, when adjacent to the azo bond, creates a fully conjugated pathway, boosting π‐conjugation and facilitating 85% *E*‐to‐*Z* and nearly 100% *Z*‐to‐*E* conversions at 400 nm and 530–550 nm, respectively. The azo photoswitches mentioned extend conjugation to redshift the π‐π* band into the visible light region, thereby achieving bidirectional visible light responsiveness. However, there is a trade‐off between the spectral red‐shifting and thermal half‐life.

Building on advances in bis‐heteroaryl azo switches, recent studies further refine these optical switches, capitalizing on the synergistic benefits of different heterocyclic moieties. Fuchter and colleagues synthesized novel visible‐light‐responsive azo photoswitches with phenyl rings substituted by benzimidazole and pyrazole rings on either side of the azo bond.^[29]^ Due to the broad pharmacological activity of benzimidazoles, this work offers insights for photopharmacology. Additionally, the pyrazole ring extends the thermal half‐life of the *Z* isomer, further contributing to this field. Li et al. developed asymmetric thiazolylazopyrazoles as bis‐heteroaryl azo switches, which utilized the thiazole's visible‐light sensitivity and the pyrazole's predisposition for facile *ortho*‐substitution.^[19]^ Notably, *ortho*‐substitution has a significant impact on the thermal stability of the *Z* isomer. As shown in Figure 6e, when there is no substitution on the pyrazole ring, the *Z* isomer is stabilized by intramolecular CHN···N bonding, with a half‐life of 2.1 days. Bis‐*ortho*‐methylation not only disrupts the CH···N hydrogen bond but also introduces steric repulsion, which reduces the thermal stability of the *Z* isomer to 1.1 days. In contrast, *ortho*‐carbonylation of the pyrazole decreased its π‐electron density, fostered a CH···π dispersion between the thiazole ring and alkyl groups as well as retained the CH···N bonding. This modification enabled near‐quantitative bidirectional photoisomerization under visible light and prolonged the *Z*‐isomer's half‐life to several days. These results demonstrate that the introduction of intramolecular attractions is a promising strategy for enhancing the *Z*‐isomer stability of visible‐light azo photoswitches.

## 3 APPLICATIONS IN ENERGY STORAGE

The azo molecule, upon light absorption, transits to its *Z* isomer, acts as an energy reservoir that converts absorbed light energy into chemical energy and stores the energy difference between the *Z* and *E* states, which is released as heat during the *Z*‐to‐*E* isomerization. This integration achieves the interconversion among light, chemical, and thermal energies, seamlessly combining solar energy absorption, conversion, thermal storage, and controllable release. Recent rapid advancements in photochemistry and energy materials have indeed been driven by a multitude of research efforts.[^54^, ^55^, ^56^, ^57^, ^58^, ^59^]

As is known to all, only 7% of the solar energy impacting Earth's surface resides in the ultraviolet spectrum, while the visible and infrared portions account for substantial fractions at 43% and 50%, respectively.^[60]^ However, visible light typically induces the reverse isomerization of azobenzene and is often used to trigger heat release. While significant progress has been made with visible light‐responsive azo small molecules, achieving both high energy density and a long half‐life in storage materials remains challenging. This is because the redshift in the molecular absorption spectrum generally compromises storage energy density.^[58]^ For instance, *ortho*‐substituted azo derivatives exhibit an energy density around 20–30 kJ·mol^−1^, notably less than the original azobenzene (41 kJ·mol^−1^).^[57]^ Therefore, developing azo‐based energy storage materials that can cover the entire solar spectrum presents a major yet essential challenge. To ensure a clear narrative development, the examples listed are not exclusively focused on storing visible light energy, but all provide insights and lay the groundwork for the field.

### 3.1 Enhancing isomerization energy

Azo benzenes and their derivatives, as light energy storage molecules, have limited energy storage density due to the small energy difference between their isomeric forms. Researchers aim to enhance the energy density and half‐life in azo‐based materials. One strategy is to covalently graft azo compounds onto nanocarbon materials or polymers, or to introduce hydrogen bonds, coordination bonds, and other interactions. This approach increases the energy barriers between the *cis* and *trans* configurations, thereby improving the energy storage density.

**FIGURE 7:**
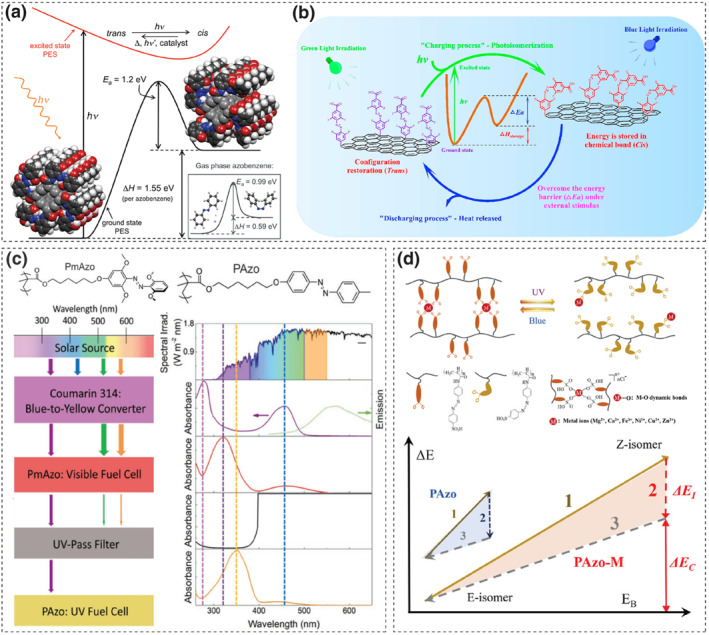
(a) The calculated energy profile between SWNT‐templated azobenzene and untethered azobenzene molecules (White, gray, blue, and red spheres represent H, C, N, and O atoms, respectively; nanotube carbons are a lighter gray). Reproduced with permission: Copyright 2011, American Chemical Society.^[61]^ (b) Schematic demonstration of the AzoTF‐rGO composite for photothermal conversion and storage. Reproduced with permission: Copyright 2021, Elsevier.^[63]^ (c) Schematic and Spectra of a 4‐Layer UV–vis Solar Thermal Device. Reproduced with permission: copyright 2021, John Wiley and Sons.^[64]^ (d) Chemical structure, schematic illustration, and energy diagram of PAzo‐M with dynamic bonds. (1) *E*‐to‐*Z* isomerization, (2) *Z*‐to‐*E* isomerization, (3) Bond enthalpy release by forming dynamic bonds or H‐bonds. Reproduced with permission: Copyright 2022, Hui Wang et al., published by John Wiley and Sons.^[65]^

Kolpak and Grossman's theoretical work demonstrated that utilizing rigid, lightweight single‐walled carbon nanotubes (SWCNTs) as templates for the ordered assembly of azo molecules under high grafting densities enhances intermolecular interactions.[^61^, ^62^] This enhancement results in a widened energy gap between *cis* and *trans* isomers and raises the barrier to *cis*‐form thermal reversion (Figure 7a). The Grossman team then produced SWNT‐Azo materials with a 1:18 grafting ratio, observing an energy density rise from 58 kJ·mol^−1^ of pure azobenzene to 120 kJ·mol^−1^, which thus validated the earlier notion.^[66]^

Feng and colleagues used reduced graphene oxide (rGO) as a carbon template, and by controlling the molecular structure and grafting density of azobenzene, constructed a series of rGO‐Azo molecular photothermal energy storage materials (MOST).[^67^, ^68^, ^69^, ^70^, ^71^] Graphene's unique lamellar structure facilitates hydrogen bonding between adjacent azobenzenes on the same or different nanosheets, which increases the isomerization energy barrier to 192.4 kJ·mol^−1^.^[68]^ Additionally, introducing three azobenzene units into a single molecule lowered the *trans* isomer's energy level and stabilized the *cis* isomer due to steric hindrance, which resulted in boosting the energy density to 943.0 kJ·mol^−1^.^[71]^ Building on this foundation, Yang et al. grafted *ortho*‐tetra‐fluorinated azobenzene onto reduced graphene oxide (rGO) molecules, to develop a material AzoTF‐rGO which can store visible light energy at low temperatures (Figure 7b). They fully exploited the photoresponsive properties of this classic molecular structure, which enabled the material to not only undergo configurational changes under visible light but also to possess a favorable storage half‐life of 80.3 h. The strategy of covalent grafting with rGO also enhanced the energy storage density of the material to 345.8 kJ·kg^−1^.^[63]^ However, these materials have a limitation due to the strong intermolecular interactions that impede the isomerization reaction. They exhibit slow photothermal energy storage, typically requiring 12 h to complete the energy storage process, and the temperature increase during the heat release is minimal.

Compared to grafting azobenzene onto carbon materials, azopolymers photothermal energy storage materials can readily form flat or thickness‐controllable films. This is crucial for efficient light capture under solid‐state conditions and for large‐area heat release applications.[^55^, ^72^] Wu and co‐workers designed an azopolymer device that could operate under sunlight, as illustrated in Figure 7c.^[64]^ The azopolymers PmAzo and PAzo were synthesized using *ortho*‐methoxy‐azobenzene and *para*‐methyl‐azobenzene as responsive molecules, respectively. The device, arranged from top to bottom, comprises coumarin 314 that transforms blue light into green‐yellow, PmAzo for visible light storage, an ultraviolet filter, and PAzo for UV energy storage. The use of two filtering layers effectively modifies the incident light, enhances the utilization efficiency of the solar spectrum and overcomes the limitation of most azobenzene photothermal materials, which solely store UV light energy. However, the energy storage density of the device is low, with energy densities of 12.2 kJ·mol^−1^ (14.4 J·g^−1^) for the PmAzo film and 54.8 kJ·mol^−1^ (125.2 J·g^−1^) for the PAzo film, which cause an overall solar capture efficiency of just 0.4% for the device.

Yet another method involves combining azobenzene molecular switches with dynamic covalent bonds or light‐triggered coordination bonds. Compared to the energy stored during the isomerization of azobenzene molecules, the enthalpy changes associated with the formation and breaking of dynamic bonds are significantly higher.[^65^, ^73^, ^74^] More importantly, these covalent bonds share many similarities with azo‐photoswitches, such as excellent reversibility and cyclicity, providing an opportunity to incorporate them into azobenzene derivatives and create MOST systems with higher energy storage densities. Feng et al. designed a series of sulfonic‐grafted azopolymers (PAzo‐M), where the dynamic bond component was formed by the interaction between sulfonate groups and various metal ions. Among them, the coordination bond formed with Mg^2^⁺ achieved both a significant energy level difference and a high degree of isomerization (86.3%), resulting in the most superior light‐to‐heat storage performance at 408.6 J·g^−1^ (113.5 W·h·kg^−1^).^[65]^ As shown in Figure 7d, under UV irradiation, PAzo‐Mg experienced *E*‐to‐*Z* isomerization and dynamic bond dissociation, simultaneously stored energy. Subsequently, the *cis*‐PAzo‐Mg absorbed blue light (450 nm) and underwent reverse isomerization, with dynamic bonds reforming, accompanied by thermal relaxation. The flexible fabric obtained by combining PAzo‐Mg with nylon fabric can release heat and provide a temperature increase of 7.7–12.5°C in outdoor low‐temperature environments (−5 to 5°C).

Enhancing the isomerization energy of azo‐based materials depends on increasing the energy gap between the *trans* and *cis* isomers. The three approaches presented in this section are primarily designed to achieve this objective. However, it is important to note that the configurational transitions in these materials are often constrained by the solid‐state structure, which makes the charging process difficult and prevents the achievement of theoretical energy storage densities. Strategies to overcome these limitations would significantly advance the use of azo photoswitches in solid‐state energy storage devices.

### 3.2 Incorporating phase change properties

**FIGURE 8:**
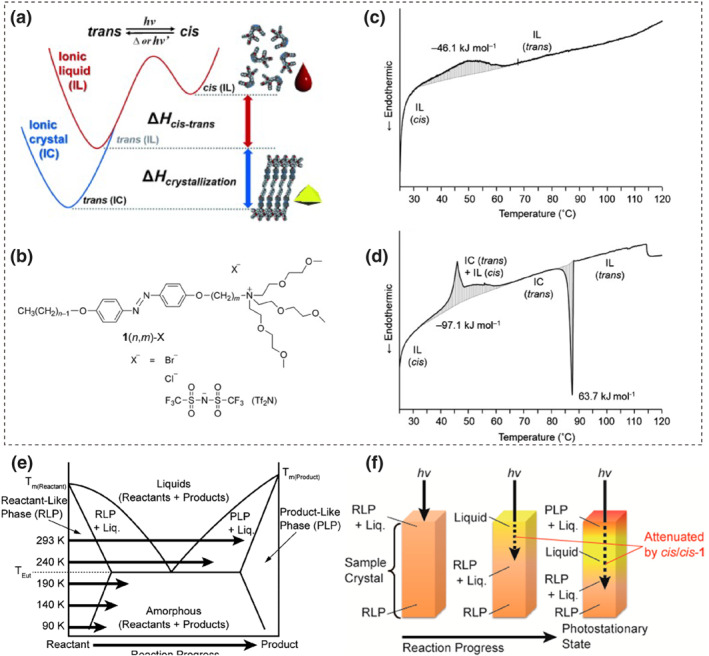
(a) Energy storage mechanism of photoliquefiable azo compounds. (b) Chemical structure of 1(n,m)‐X. DSC thermograms of *cis*‐azobenzene derivatives: (c) *cis*‐1(6,4)‐Tf_2_N (IL) and (d) *cis*‐1(6,4)‐Br. Reproduced with permission: copyright 2014, John Wiley and Sons.^[75]^ (e) Two‐component phase diagram of azobenzene derivatives. (Molecules prior to UV illumination are referred to as reactants, and those after UV radiation are denoted as products.) (f) Schematic of photoliquefaction. Reproduced with permission: Copyright 2014, American Chemical Society.^[76]^

In 2015, the Morikawa group first proposed a novel strategy to enhance the energy storage density of azo‐based solar fuels: integrating the *trans*‐to‐*cis* isomerization of azobenzene with photoinduced solid‐liquid phase transitions. During the energy release, the *cis*‐to‐*trans* isomerization releases heat, accompanied by an additional phase change enthalpy as the liquid transits back to a crystal (Figure 8a).^[75]^ In this study, Morikawa et al. developed ionic crystals (ICs) of a cationic azobenzene derivative, as shown in Figure 8b. These molecules have long alkoxy chains and oligo‐ (ethylene oxide) units at both ends of the azobenzene, which can lower the melting point (*T*_*m*_) of the molecules. Replacing the anions from Tf_2_N^−^ with Br^−^ and Cl^−^ further reduces the *T*_*m*_. Figures 8c,d show the DSC curves for the *cis*‐azo ionic liquids. It noticed that *cis*‐1(6,4)‐Tf_2_N^−^ ionic liquid exhibits only one exothermic peak (corresponding to *cis*‐to‐*trans* isomerization), whereas *cis*‐1(6,4)‐Br^‐^ ionic liquid displays two overlapping exothermic peaks, including the *cis*‐to‐*trans* isomerization exotherm and a crystallization exotherm from the ionic liquid to the ionic crystal. The combined heat of these two processes reaches 97.1 kJ·mol^−1^, which is twice the heat of isomerization alone. This demonstrated that the light‐induced phase transition can be used to store photon energy, thereby refreshing the upper limit of azobenzene energy storage capacity.

Due to its large molecular weight, the weight‐specific energy density of this compound is only 128 J·g^−1^, which is insufficient for practical applications. However, the underlying concept remains intriguing and promising. Researchers have begun to increase the energy storage density of azo materials by incorporating phase change properties.

In fact, as early as 2012, Okui and Han observed the photomelting phenomenon of azobenzene using optical microscopy, but did not conduct further investigation.^[77]^ For solid‐state azobenzene, under appropriate wavelength light stimulation, the *trans* form on the surface converts to the *cis* form, which results in altering the molecular arrangement within the phase domains during this transformation. Since the *trans* isomer typically has a rod‐like shape with high order and strong intermolecular forces (such as π‐π stacking), the *T*_*m*_ of *trans* crystals is usually above room temperature, which prevents them from melting at room temperature. Upon illumination, a significant portion of the *trans* isomers convert to the bent *cis* isomers, which decrease the molecular packing density and weaken intermolecular forces. This change, contributes to a reduction in the melting point of the material.[^78^, ^79^] When the *T*_*m*_ of the azo small molecule shifts from above room temperature to below room temperature upon illumination, room‐temperature light‐induced solid‐to‐liquid phase transitions can occur.

In most cases, the two isomers coexist as a mixture in a certain ratio under photo‐stationary state (PSS) conditions, so the observed photomelting phenomenon is actually the melting of the mixture. Furthermore, numerous researchers have demonstrated that the *T*_*m*_ of a mixture of *trans* and *cis* form in certain proportions can be significantly lower than that of pure *trans* or *cis* form.[^80^, ^81^] For systems containing a mixture of *trans* and *cis* isomers, the relationship between the system's melting point and the ratio of the two isomers can be represented by a phase diagram, as shown in Figure 8e.^[76]^ Therefore, as long as the eutectic temperature is below room temperature, light‐induced solid‐to‐liquid transitions can be achieved even if the melting points of both the *trans* and *cis* isomers are above room temperature.

In addition to leveraging the phase change properties to enhance the overall energy storage capacity of the system, the advantage of optically controlled phase transition azo‐based materials in energy storage also lies in improving the ability of underlying layers of azobenzene to absorb photons for energy storage, as illustrated in Figure 8f.^[76]^ As the surface layer of azobenzene undergoes isomerization to store energy and liquefies, the liquid phase experiences reduced reflection and scattering of photons, and as well has a tendency to flow outward, which allows photons to penetrate deeper into the material until the PSS is reached. Some photons are attenuated due to the high molar extinction coefficient of azobenzene, which can cause incomplete phase change at the bottom when the PSS is achieved.^[82]^ Despite of its negative impact, the absorption capacity for photons is still significantly improved compared to the system without phase change.

#### Azo small molecules

The Norikane group has proposed several strategies for designing azo small molecules that can undergo optically controlled phase transitions.[^82^, ^83^, ^84^, ^85^, ^86^, ^87^] Among these, the design concept of asymmetrically substituted alkoxyl azobenzene is particularly important. Lowering molecular symmetry facilitates isomerization and weakens intermolecular interactions in the *cis* isomer, which leads to azo small molecules with lower melting points.^[88]^ Researchers have designed a series of such azo molecules and utilized them as energy storage materials.

##### Azobenzene derivatives

**FIGURE 9:**
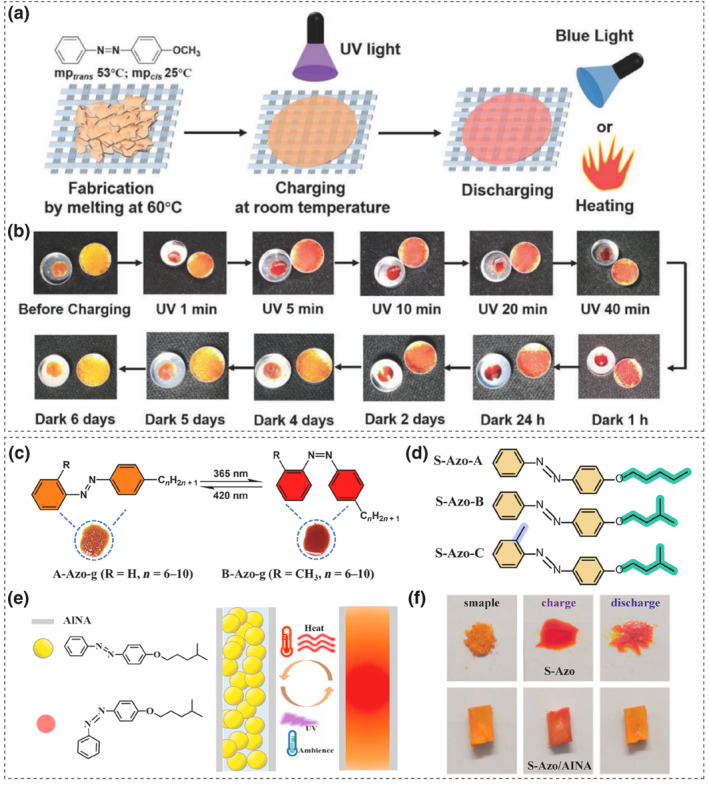
(a) Fabrication of Azo‐OMe/fabric composite (b) Charging and discharging of Azo‐OMe and Azo‐OMe/fabric composite. Reproduced with permission: copyright 2019, John Wiley and Sons.^[89]^ Chemical structure of (c) two Azo‐g and (d) three S‐Azo molecules. Reproduced with permission: Copyright 2022, Jian Gao et al., published by John Wiley and Sons. (e) Schematic illustration of energy storage and release of S‐Azo/AlNA. (f) Morphology of charged and discharged S‐Azo and S‐Azo/AlNA at 25°C. Reproduced with permission: Copyright 2021, Elsevier.[^90^, ^91^, ^92^]

Yu and colleagues selected a simple yet classic azo crystal, 4‐methoxyazobenzene (Azo‐OMe), as an energy storage material. The melting points of the *trans* and *cis* isomers of Azo‐OMe are 53°C and 25°C, respectively. At room temperature, Azo‐OMe can undergo a photoinduced crystal‐liquid phase transition upon UV light exposure. As the liquefied material loses its solid‐state properties, a flexible fabric template was introduced to adsorb the liquid Azo‐OMe between the fibers' large gaps to prevent leakage (Figure 9a,b). The energy storage density of the composite material reaches 202 J·g^−1^, and the temperature difference between the charged and discharged samples under blue light stimulation (460 nm) is around 4°C, with a half‐life of 1–2 days^[89]^ Integrating photo‐induced phase‐change azobenzene derivatives with commercial fabrics results in a flexible solvent‐free solid‐state energy storage system. This system directly stores and releases energy, better meeting practical application needs.

Subsequently, the phase change energy storage systems based on azo molecules were extensively studied and analyzed. Han et al. found that the size and polarity of the *p*‐substituents attached to azobenzene significantly affect the phase of the isomers and their energy storage capacity.^[93]^ Feng et al. elucidated the impact of alkyl chain length on the energy storage properties of phase change azo photoswitches. The relatively short‐chain alkyl groups feature weaker molecular interactions, which facilitate the solid‐liquid phase transition (i.e., lower crystallization temperature *T*_*c*_ for the *cis* isomer), however result in a lower energy storage density.^[94]^ Griffiths et al. attempted to mix azobenzene **a** with 4‐methoxyazobenzene **b**. Upon irradiation, the four‐component mixture of *E*‐**a**, *Z*‐**a**, *E*‐**b**, and *Z*‐**b** can further reduce the melting point, thereby enabling photomelting at temperatures below the melting point of a single component. This allows the operational temperature range of the azo‐based energy storage systems to be extended from −58°C to 31°C.^[95]^

Feng and colleagues designed and synthesized two asymmetric structures of alkyl‐grafted azobenzenes. The study controlled the crystallization and isomerization of the azo molecules (Azo‐g) by adjusting the length of the alkyl chains and the methyl groups (Figure 9c).^[90]^ It was observed that the introduction of side groups significantly lowers the compound's melting point. This is because the side substituents reduce the structural symmetry and thus the compactness of molecular packing, which facilitate the occurrence of phase transitions. As a result, Azo‐g can store both crystallization and isomerization enthalpies over a wide temperature range (−79 to 25°C) and achieve a high energy release of 275–303 J·g^−1^. Notably, it can enable controllable thermal energy release (>300 J·g^−1^) at extremely low temperatures (−79°C), which fulfills the application needs of energy storage materials under extreme conditions.

Similarly, they designed three asymmetric alkoxy‐substituted azobenzene (S‐Azo) with similar molecular weights to further investigate the effects of substituents on isomerization and crystallization, as shown in Figure 9d.^[91]^ N‐alkoxy substitution endows S‐Azo with strong van der Waals forces, which facilitates crystallization but hinders isomerization. Branched alkoxy chains disrupt π‐π stacking and weaken the van der Waals forces between alkoxy groups, which increase the degree of isomerization and thus boost the isomerization enthalpy. But this comes at the expense of crystallization enthalpy. Therefore, there is a trade‐off between crystallization and isomerization that must be considered in practical applications.

Subsequently, S‐Azo‐B was encapsulated in aluminum nitride airgel (AlNA) to fabricate an energy storage device (Figure 9e,f). Porous airgel design is attracting attention in the field of energy storage due to their high specific surface area, high porosity, and low thermal conductivity.^[96]^ The interlayer porous structure of the airgel ensures appropriate light transmittance and good interface compatibility with the azobenzene compound. The composite device can release thermal energy and produce a temperature difference of 1.5°C with 420 nm light.^[92]^

The aforementioned work synthesized azobenzene derivatives with light‐induced phase change characteristics. The liquid azo molecules provide a more free volume for isomerization, thereby increasing the isomerization rate. This addresses the issue of solid azobenzene being limited by steric hindrance and requiring solvents for isomerization charging. Furthermore, the challenge of slow heat release is mitigated by their solid‐liquid phase change functionality. However, the fluidity during the phase change process can disrupt the original structure of the system. Therefore, using porous flexible materials as templates to create solid‐state energy storage devices is a design approach with greater practical application potential.

##### Azoheteroarenes

**FIGURE 10:**
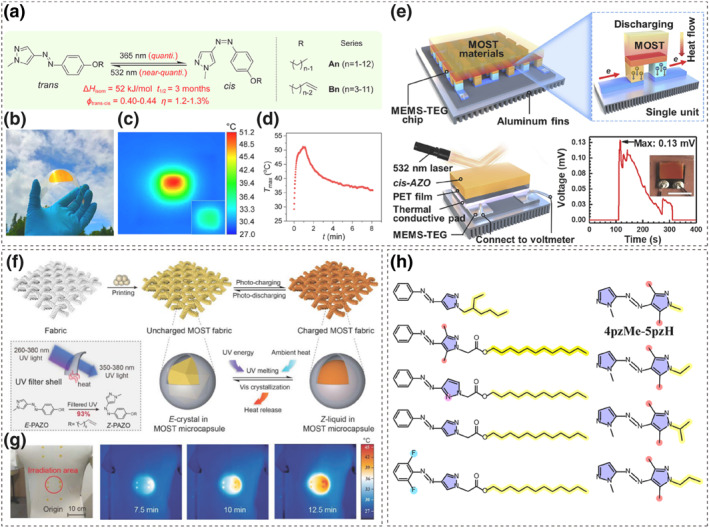
(a) Structure and photochemical properties of pzAzo ethers. (b) Photograph of a flexible film device. (c) IR thermal images of the film during photodischarging taken at the highest *T*_max_. (d) *T*_max_ as a function of time during 532 nm light irradiation of the film. Reproduced with permission: Copyright 2020, American Chemical Society.^[97]^ (e) Schematic of the integrated generator device. Structural schematic of the device enabled by photoliquefiable arylazopyrazole. Net voltage generated from the device chip over time. Reproduced with permission: Copyright 2022, Zhihang Wang et al., published by Elsevier.^[98]^ (f) Illustration of the molecular solar thermal (MOST) fabric. (g) Heat release at specific acupoints upon 532 nm light irradiation. Reproduced with permission: copyright 2023, John Wiley and Sons.^[99]^ (h) Structures of arylazopyrazole derivatives exhibiting photoliquefaction.

Li and colleagues developed a series of pyrazolylazophenyl ether‐based azo molecules (pzAzo ethers) with long linear alkyl chains. By adjusting the molecular structure, some of these molecules are capable of reversible photochemical phase transitions. These key structure modifications include but not limited to changing the number of carbon atoms in the alkyl chain or substituting the terminal group (alkyl or vinylic end group), as shown in Figure 10a.[^97^, ^100^, ^101^] In particular, the high isomerization ratio and long half‐life of the pzAzo ethers were leveraged to achieve efficient and long‐term photothermal energy storage. The system achieved an energy storage density of 330–370 J·g^−1^, and the energy storage half‐life at room temperature was extended to three months. Further fabricated flexible film devices could release heat exceeding 20°C above room temperature (Figure 10b‐d).^[97]^

This flexible device not only absorbs photon energy but also spontaneously absorbs ambient heat, which is difficult to utilize efficiently. This absorbed heat overcomes the molecular packing in the crystal, generating *cis* liquid and enabling the simultaneous storage of both light and thermal energy. This significantly increases the energy storage density. However, the narrow range of absorbed photon energies limits its practical application.

In addition, Li et al. used the prepared azo films for solar thermal electrical power generation devices (Figure 10e). Thermoelectric generators (TEG) can directly convert thermal energy into electrical energy via the Seebeck effect. By combining arylazopyrazole derivative‐based MOST films with microelectromechanical system‐based thermoelectric generators (MEMS‐TEG), solar energy can be stored and converted into electricity. Although the efficiency of the device needs improvement (maximum power output density of 1.3 W·m^−^³), this study presents broad prospects for the preparation and application of azo‐based energy storage devices.^[98]^

Following this, they collaborated with Fei et al. to construct UV‐filtering microcapsules. These microcapsules used the pyrazole‐based azo molecules as the core material, while the shell was composed of the UV‐absorbing component 4‐benzoyl‐3‐hydroxyphenyl acrylate, and high light‐transmittance polystyrene.^[99]^ Microencapsulation technology has been widely applied to traditional phase change materials (PCMs), which enhance melting/crystallization kinetics by increasing specific surface area.^[102]^ The UV‐filtering microcapsules they prepared can filter out wavelengths in the deep UV region (260–350 nm) that are detrimental to the isomerization of azo molecules. This approach allows azo molecules to maintain a high isomerization conversion rate even under broad‐spectrum UV light (260–380 nm), while also addressing the issue of leakage during the phase change process. Printing microcapsules onto the surface of fabrics produces high‐energy‐density pyrazole‐based azo molecular solar thermal fabric. The surface temperature of these textiles can reach up to 46°C during energy release. Leveraging the high‐contrast photochromic performance of azopyrazole, visual energy indicators can be realized. Their application in targeted acupuncture points for solar thermotherapy further expands the use of azo photoresponsive materials in personal health care, achieving efficient personal thermal management (Figure 10f,g).

As shown in Figure 10h, Morikawa and colleagues introduced a 2‐ethylhexyl group onto the pyrazole ring of arylazopyrazole, resulting in a molecular solar thermal fuel with a half‐life of 3069 h.^[103]^ Gerkman et al. used arylazopyrazole derivatives as core structures and functionalized them with fatty acid moieties, enabling the molecules to exhibit photoliquefaction.^[104]^ Notably, the designed azo molecules possess supercooling crystallization capabilities, allowing the *cis* form to maintain liquid‐phase stability even at −33°C. This improvement prevents the uncontrolled crystallization of the *Z* isomer at low temperatures, thus preserving its latent heat storage capacity.

In recent years, the design and synthesis of bis‐heteroaryl azo compounds have garnered significant attention. Fuchter et al. developed photo‐controlled energy storage materials based on azobispyrazoles. As shown in Figure 10h, by using 4pzMe‐5pzH as the core structure, they varied the terminal alkyl group on the N atom of the 4pzMe ring to influence the molecular packing of the *E* and *Z* isomers, thereby modulating their *T*_*m*_.^[105]^ Of particular note is that as the compound gradually liquefies, the molecular diffusion and convection within the liquid can help enhance the conversion rate from *E* to *Z* isomer. Compared to typical photoswitches with long alkyl chains, azobispyrazoles with small substituents demonstrated an effective light penetration depth over 1400 μm due to reduced van der Waals interactions.

Azo molecules containing heterocycles exhibit different properties compared to azobenzene derivatives. Particularly, arylazopyrazoles are favored for their high bidirectional isomerization ratio or long thermal half‐life of the *Z* isomer. When endowed with phase change characteristics, their energy storage properties are further improved. These heterocyclic azo photoswitches hold promise as alternatives to traditional azo photoswitches and expand the variety of azo‐based energy storage materials.

##### Visible‐light‐responsive azo molecules

It can be concluded from the above discussion that effective phase transition strategies have been widely applied in azo photoresponsive materials. Storing both isomerization enthalpy and phase transition energy in the visible light range is also highly attractive. Combining the design strategies for visible‐light‐responsive azo molecules (as detailed in Chapter 2) with those for light‐induced phase transition presents a promising approach.

**FIGURE 11:**
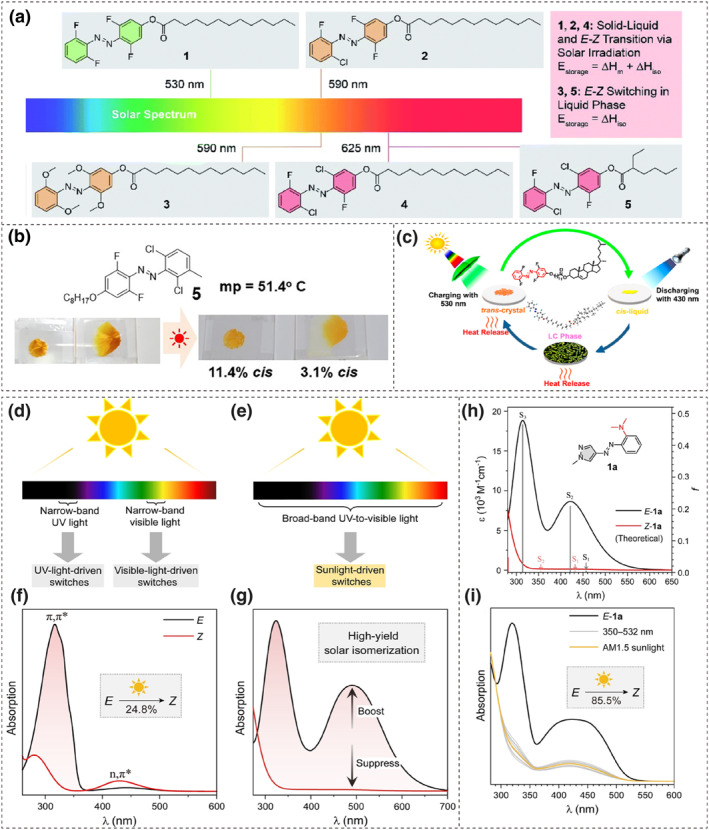
(a) Chemical structures of azobenzene derivatives that could store photons in the visible light range. Reproduced with permission: Copyright 2021, Royal Society of Chemistry.^[106]^ (b) Appearance of a photoliquefiable azobenzene derivative before and after sun exposure. Reproduced with permission: Copyright 2023, Dennis Kwaria et al., published by American Chemical Society.^[107]^ (c) Schematic of the photomelting process of an azobenzene‐derived liquid crystals. Reproduced with permission: Copyright 2021, American Chemical Society.^[108]^ Comparative illustrations of common switches driven by (d) narrow‐band lights and (e) broad‐band lights. (f) Experimental UV‐vis absorption spectra of azobenzene. (g) Illustration of the spectral reshaping strategy for high‐yield solar *E*‐to‐*Z* isomerization of azo‐switches. (h) Theoretically predicted and (i) experimental UV‐vis absorption spectra of **1a**. Copyright 2024, Tao Li et al., published by John Wiley and Sons.^[109]^

As shown in Figure 11a, Han et al. designed a series of azo energy storage materials that undergo solid‐to‐liquid phase transitions under filtered sunlight. They used visible‐light‐triggered *ortho*‐substituted azobenzene derivatives as head groups and tridecanoic or ethylhexanoate esters as tail groups, which resulted in a lowered melting temperature.^[106]^ In particular, when using an *o*‐fluoroazobenzene derivative as the head group, the compound exhibits a long‐term storage half‐life (2 months) over a wide temperature range (−40°C–110°C) and demonstrates high photoisomerization efficiency (*trans* → *cis* ≈ 91%, *cis* → *trans* ≈ 81%). Its energy storage density was approximately 70 kJ·mol^−1^ (including 25 kJ·mol^−1^ for isomerization enthalpy and 45 kJ·mol^−1^ for phase transition enthalpy), which was sufficient to raise the sample temperature by 2–3°C during energy release. Similarly, Norikane et al. employed *ortho*‐halogen‐substituted azobenzene derivatives as head groups and introduced alkoxyl chains at the *para*‐position to facilitate visible light‐induced solid‐to‐liquid phase transitions. The synthesized azo compounds could even be photomelted directly by sunlight (Figure 11b).^[107]^

Building on this, Gupta et al. designed visible‐light‐responsive solar‐thermal fuels (STFs) based on liquid crystals (LCs), consisting of *o*‐fluoroazobenzene derivatives and cholesteryl groups (Figure 11c). This LC system undergoes a phase transition from the liquid crystalline phase to an isotropic liquid during photoisomerization, which is manifested as a distinct extinction phenomenon under polarized microscopy. During energy release, the maximum temperature rise relative to the surrounding environment can reach 5.4°C, which is higher than that of similar azobenzene derivatives lacking the liquid crystalline phase.^[108]^

Li and colleagues introduced a stronger *p*‐electron‐donating group, the 4‐thioalkyl group, to modify the arylazopyrazoles, which resulted in a redshift of the maximum absorption wavelength to achieve bidirectional visible light‐driven isomerization. Through appropriate structural design, molecules capable of capturing and releasing both phase change and isomerization energy at temperatures below 0°C were developed.^[110]^ These designs include changing the bridging positions between the azo group and the pyrazole ring, and varying the length of linear alkyl chains with or without a vinylic end group on the thioalkyl group. The qualified molecule was mixed with functionalized SiO_2_ to prepare a leak‐free energy storage coating, which was then applied on glass to simulate an intelligent heat storage window. This coating can be charged and discharged under visible light (charged with 400 nm light and discharged with 532 nm light) at an environmental temperature of −1°C, with the surface temperature increasing by 22.7°C within 60 s during discharge.

However, the visible‐light switches typically need to operate within specific narrow visible windows, which makes it challenging to effectively utilize solar energy directly (Figures 10f and 11d). Li et al. proposed a novel molecular design strategy where *E* isomer shows a strong absorption band across a broad spectral range of 260–600 nm, while the absorption of the *Z* isomer becomes negligible. The strong absorption of the *E* isomer relative to the *Z* isomer across this broad spectrum ensures that the *E*‐to‐*Z* isomerization process dominates under direct sunlight exposure (Figures 11e,g). The proportion of the *Z* isomer in the photo‐stationary state (PSS) is determined by the ratio of the absorbances of the two isomers. The design of molecule structure **1a** validated the aforementioned strategy (Figure 11h,i). It inherits the symmetrical T‐shaped geometry of the *Z* isomer from the parent phenylazopyrazole, which suppresses its n→π* transitions. Benefitting from the spatial overlap between the π_*n*_ orbital (mainly localized on the dimethylamino‐phenyl fragment) and the π* orbital at the benzene ring, the π_*n*_→π* transition is symmetry‐allowed, which results in a strong visible light absorption band (S_2_).^[109]^ This enables it to produce 85.5% of the *Z* isomer under unfiltered light conditions, with a long thermal half‐life of 96 days. Additionally, the reverse *Z* to *E* process can be achieved by introducing a *para*‐methoxy group to tune the n→π* transitions in molecule **1a**. This innovative molecular design opens up new opportunities for the application of azo compounds in solar energy technologies.

#### Azopolymers

Unlike small azo molecules, azopolymers do not typically exist in crystalline forms but rather in amorphous states or exist in a combination of amorphous and crystalline regions. High molecular weight polymers may not provide sufficient free space for isomerization due to significant chain entanglements and limited segmental mobility.^[111]^ Therefore, it is crucial to fully utilize the amorphous regions in azopolymers to provide sufficient freedom for the photoisomerization. Research results indicate that the glass transition temperature (*T*_*g*_) of azopolymers determines their state at room temperature, with *T*_*g*_ typically varying with structural changes. This provides theoretical guidance for the reversible phase transitions of azopolymers. Incorporating azo units into polymers, the photoisomerization from *trans* to *cis* leads to a drastic change in molecular shape. Notably, the *cis* isomer often exhibits weaker intermolecular π‐π stacking interactions and reduces the molecular regularity, which ultimately overcomes the frozen chain segments and significantly decreases the *T*_*g*_.[^78^, ^79^, ^81^] Similar to small azo molecules, azopolymers do not necessarily require 100% of the *cis* isomer; photomelting can occur as long as the proportion of the *cis* isomer reaches a certain range.[^112^, ^113^, ^114^]

**FIGURE 12:**
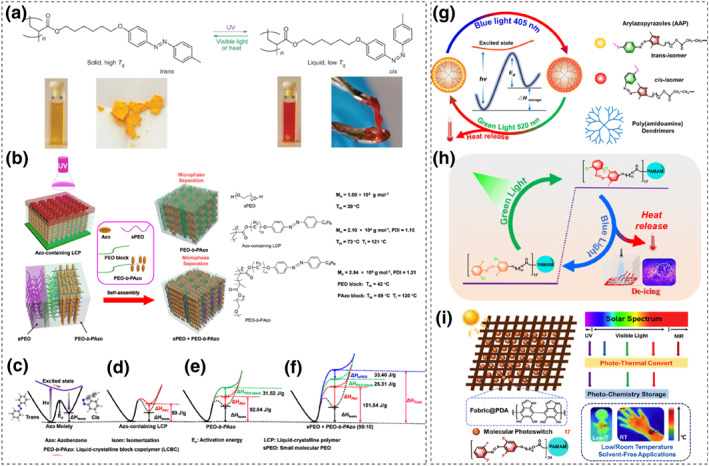
(a) Chemical structure and photographs of an azopolymer with an azobenzene group in the side chain. Reproduced with permission: Copyright 2016, Springer Nature.^[120]^ (b) Illustrations of Azo‐containing LCP upon UV irradiation, microphase separation of PEO‐b‐PAzo, and the self‐assembly of sPEO and PEO‐b‐PAzo composites. Solar thermal energy storage within (c) azo moiety, (d) Azo‐containing liquid‐crystalline polymers, (e) PEO‐b‐PAzo block copolymer, and (f) composite of sPEO and PEO‐b‐PAzo. Reproduced with permission: Copyright 2021, American Chemical Society.^[122]^ Schematic diagram of (g) AAP‐ and (h) FClAzo‐containing dendrimers absorbing photon energy and heat release. Reproduced with permission: Copyright 2021, American Chemical Society.^[123]^ Reproduced with permission: Copyright 2024, Royal Society of Chemistry.^[52]^ (i) Schematic diagram of the composition of the flexible wearable fabrics and the solar energy harvesting and storage in various light environments. Reproduced with permission: Copyright 2023, Elsevier.^[124]^

In recent years, an increasing number of azopolymers with sufficient free volume and segmental mobility have been synthesized, demonstrating that the *T*_*g*_ of azopolymers can be modulated by light stimulation, leading to reversible solid‐to‐liquid transitions.[^115^, ^116^, ^117^, ^118^, ^119^, ^120^, ^121^] Wu et al. synthesized a polyacrylate polymer with azobenzene in the side chain, which has a number‐average molecular weight of 9900 g·mol^−1^ (Figure 12a). The *T*_*g*_ of its *trans*‐Azo polymer is above 48°C, while the *cis*‐azo polymer's *T*_*g*_ is below ambient temperature (about −10°C).^[120]^ Thus, this system can undergo a light‐induced phase transition at room temperature. Although the energy storage properties of these azopolymers were not the focus of this work, it provides a valuable approach for understanding and designing phase change azopolymer materials. However, it should be noted that the inferior flowability and transparency of polymer melts after phase transition, compared to liquids, means that photoinduced phase changes in bulk polymers essentially occur only on the surface. Moreover, solvent assistance is often required when measuring *T*_*g*_.

Cai et al. prepared liquid crystalline block copolymers (LCBCs) using the light‐responsive azobenzene liquid crystal polymer PAzo as the continuous phase and polyethylene oxide (PEO, ca. 5 kDa) as the dispersed phase. These approaches enhanced the energy storage density by adding low‐molecular‐weight PEO (sPEO, ca. 1 kDa). As shown in Figure 12b, the sPEO, serving as a phase change material, could be uniformly dispersed in the PEO‐b‐PAzo block copolymer without disrupting the original microphase separation (MPS) structure.^[122]^ The nanocolumnar structure formed by MPS confines the melted sPEO and PEO blocks at the nanoscale, resulting in a significant decrease in the sPEO crystallization temperature (from 24°C to −38°C), which preserve the energy storage capability of the sPEO and PEO blocks at low temperatures. The LCBCs can also simultaneously store both the isomerization energy and the phase transition energy from the liquid crystalline phase to the isotropic phase under UV light. The prepared composite film (sPEO + PEO‐b‐PAzo) achieved an energy density of 210.3 J·g^−1^, with the released energy comprising all four components, as shown in Figure 12c–f. This composite material can be utilized to fabricate insulating textiles, which are capable of increasing the fabric surface temperature from 32.2°C to 43.6°C within 180 s.

In contrast to ordered structures, disordered structures, such as dendritic architectures, exhibit weaker interactions between macromolecular chains and azobenzene derivative molecules. This results in lower energy storage densities but typically faster isomerization rates, which have attracted attention from some researchers. Xu et al. covalently grafted azobenzene acrylate derivatives onto polyamide‐amine (PAMAM) dendrimers, which contain a high density of reactive groups, to prepare dendritic photothermal energy storage materials.^[125]^ Importantly, upon exposure to ultraviolet light, the powdered dendritic polymer transforms into a red viscous liquid, which subsequently reverts to a solid state under visible light irradiation. The abundant reactive groups on the surface of PAMAM dendrimers create conditions for the high‐density grafting of azobenzene photostorage units, resulting in a polymer with an energy storage density of up to 212.4 J·g^−1^ (59 W·h·kg^−1^).

Building on this foundation, Xu et al. covalently grafted blue‐light‐responsive arylazopyrazole (AAP) acrylate derivatives and green‐light‐responsive fluoro‐chloro‐azobenzene (FClAzo) acrylate derivatives onto PAMAM dendrimers, as shown in Figure 12g,h.[^52^, ^123^] Attributed to the substantial absorbance of sulfur‐methyl‐substituted arylazopyrazole groups under visible light wavelengths and the thermal stability of their *cis* isomers, the AAP‐containing dendritic polymer film achieves an energy density of 0.14 MJ·kg^−1^ (67 kJ·mol^−1^) and a storage half‐life of up to 12.9 days upon effective collection of blue light (405 nm). Its absolute heat release temperature can reach up to 6.5°C. The FClAzo‐containing dendrimer, although achieving an energy density of only 0.046 MJ·kg^−1^ (19 kJ·mol^−1^) upon irradiation with green light (520 nm), exhibits a remarkably long storage half‐life of 20.6 days. Furthermore, due to the favorable chain mobility of the dendrimer, the release of thermal energy can elevate the temperature by 3.7°C in a cold environment (−2°C).

Similarly, the team designed the grafting of tetra‐fluoro‐azobenzene acrylate derivatives onto the surface of PAMAM dendrimers, which enables the simultaneous storage of photon energy from ultraviolet, green, and red light.^[124]^ By integrating this with fabric coated in polydopamine, they developed a flexible wearable fabric (Figure 12i). This composite fabric has an energy density of 56 J·g^−1^ (18.2 kJ·mol^−1^) and a storage half‐life of approximately 1 month. Upon triggering by blue light, it achieved a temperature increase of 11.1–12.3°C due to thermal release. These remarkable characteristics are attributed to the redshifted n‐π* band of the *o*‐fluoro‐azobenzene, the low *T*_*g*_ of the dendrimers, and the photothermal effect of polydopamine. The aforementioned work further enhances the alignment of azobenzene photothermal materials with the solar spectrum, advancing the development of molecular photothermal materials towards visible light energy storage.

Azopolymers have good processability and are suitable for constructing solid devices. Especially, azopolymers can undergo efficient photoisomerization in the solid state due to their ability to undergo photochemical phase transitions, which makes them potential candidates for solid‐state solar thermal energy storage. Some studies have tried to overcome the limitations of UV charging by designing azo groups that respond to visible light, but the energy density of the stored energy remains relatively low. Emerging strategies are still needed to address these challenges.

#### Composite systems doped with azo compounds and PCMs

Due to synergistic interactions among molecules, the photoisomerization of azo compounds not only regulates their own phase transition behavior but also acts as a photoswitch to control the phase state of non‐photoresponsive materials. Conventional phase change materials (PCMs) lack control over the storage time and have a narrow temperature range for heat storage as the reversible phase change typically occurs at fixed melting and crystallization points that are very close to each other.[^126^, ^127^] When azo compounds are combined with traditional phase change molecules, the *cis* isomer weakens the intermolecular interactions, resulting in a lowered phase transition point and widening the temperature range for heat storage. More importantly, after the *cis* to *trans* isomerization under light, the *trans* isomer acts as a nucleating agent for crystallization in PCMs, preventing uncontrollable supercooling and ensuring controlled latent heat release.

**FIGURE 13:**
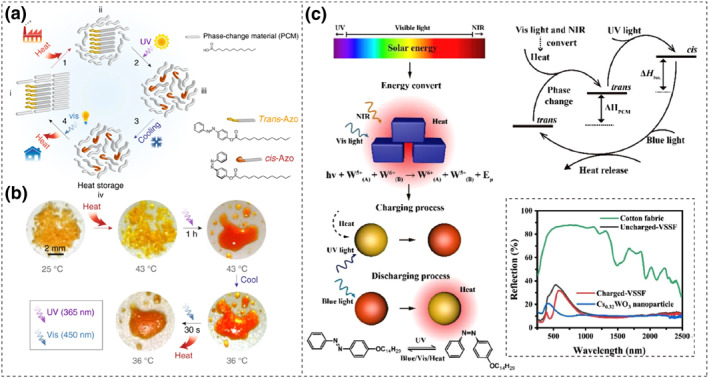
(a) Schematic and (b) real product images of tridecanoic acid and azobenzene dopants during thermal energy storage and release. Reproduced with permission: Copyright 2017, Grace G. D. Han et al., published by Springer Nature.^[128]^ (c) Schematic of UV‐Vis‐NIR spectrum solar thermal energy harvesting, storage and release. The inset shows the UV‐Vis‐NIR reflection spectra of charged‐VSSF, uncharged‐VSSF, Cs_0.32_WO_3_ nanoparticle and cotton fabric. Reproduced with permission: Copyright 2021, Elsevier.^[129]^

The Grossman research group first introduced azo molecules as dopants in the organic phase change materials, which provides a new avenue for the application of azo compounds in energy storage (Figure 13a). Azobenzene was functionalized with a tridecanoic ester group to ensure sufficient miscibility with tridecanoic acid.[^127^, ^128^] As illustrated in Figure 13b, the crystalline composite material (solid PCM + *trans*‐Azo) absorbs external heat. Upon reaching the composite's melting point (*T*_*m*_ = 43°C), it transforms into a mixture of liquid PCM and solid *trans*‐Azo. Upon UV light exposure, the *trans*‐Azo transits to the *cis*‐Azo and is well‐dispersed in the liquid PCM, which results in steric repulsion and dipole interactions that disrupt the packing of PCM molecules. As a result, the liquid composite (liquid PCM + *cis*‐Azo) remained in a liquid state even when cooled below its original crystallization temperature (*T*_*c*_ = 38°C), thus preserving the stored thermal energy. Furthermore, by adjusting the type of organic phase change material and the doping ratio of the azo molecules, the crystallization temperature can be further reduced. This adjustment allows the stored latent heat to be preserved at lower temperatures, providing valuable insights for the design of portable thermal energy storage systems.

To further expand its application scenarios, similarly, Liu et al. combined long‐chain alkyl azobenzene molecules (C_14_Azo) with the organic phase change material tetradecane to create the Azo@Tde composite material. This composite is capable of photoinducing solid‐liquid phase transitions at low temperatures (<0°C), releasing a significant amount of heat (207.5 J·g^−1^).^[130]^ Fei et al. introduced the C_14_Azo into the alkyl alcohol (C_14_OH) phase change system, where the C_14_OH acts as a “solvent” to disperse and arrange the C_14_Azo. This “solvent” effect weakens the π‐π stacking interactions between the benzene rings, ensuring rapid isomerization reactions of the azo molecules even in the solid state. Building on this, they added nano‐cesium tungsten bronze powder (Cs_0.32_WO_3_), which primarily absorbs near‐infrared and visible light, to complement the absorption range of the C_14_Azo and enhance the coverage of solar spectrum absorption. The heat generated by Cs_0.32_WO_3_ can also be stored in the phase change molecule C_14_OH as latent heat. Figure 13c illustrates the process of solar thermal energy harvesting, storage, and release. To prevent leakage during the phase change process, the C_14_OH@C_14_Azo were encapsulated in highly transparent polystyrene microcapsules, resulting in a visible solar spectrum energy storage fibric (VSSF) with a total solar conversion efficiency of 4.8%.^[129]^ The visualization is achieved by using the isomeric ratio of azobenzene derivatives as a bridge, leveraging the color change that occurs before and after isomerization to establish a correlation between energy storage density and color parameters. Although the total energy storage density of the textile (30 J·g^−1^) requires improvement, this approach provides valuable insight for the full‐spectrum solar utilization in azo‐based energy storage textiles.

## 4 CONCLUSIONS AND PROSPECTS

This review article discusses the design of visible‐light‐responsive azobenzene‐based smart materials and their applications in energy storage. Recently, there has been growing interest in azobenzene derivatives with bidirectional visible‐light switching properties. Achieving bidirectional visible‐light‐induced isomerization is particularly valuable for energy storage applications as it enhances the material's compatibility with the solar spectrum and creates an attractive pathway to improve solar energy conversion efficiency.

Azobenzene and its derivatives serve as crucial molecules for creating smart light‐responsive materials. Therefore, it is of great significance to improve their photoresponsive properties through appropriate molecular design. Challenges remain despite numerous exciting studies, particularly with the emergence of heteroaryl azo compounds, offering unprecedented photophysical properties. On one hand, there are relatively few reports on azo photoswitches activated by long‐wavelength light (red or NIR photons), and those that do exist often exhibit unsatisfactory performance in other properties such as isomer stability and isomerization efficiency. On the other hand, there is necessary demanding for further optimization of the molecular synthesis process to reduce production costs and to overcome the transition barriers from laboratory‐scale to large‐scale industrial production. To address these challenges, guiding molecular design through theoretical computing and machine learning offers a promising solution. Rational and effective theoretical predictions can reduce trial and error, thereby lowering experimental costs. Notably, machine learning has made considerable advancements in targeted molecular design in recent years.^[131]^

To address the low energy density issue of azo‐based energy storage materials, researchers have made considerable efforts for improvements. However, these strategies have limitations that require further refinement. For example, while carbon templates increase energy storage density, the aggregation of azobenzene groups within them hinders photoisomerization in the solid state. This often requires solvent‐assisted charging. Photo‐liquefiable azo derivatives can achieve solvent‐free charging and controllable thermal release via light. However, their energy storage density significantly decreases when they are bidirectionally induced by visible light. Additionally, template encapsulation is necessary to prevent liquid leakage.

Besides energy storage density, factors such as storage half‐life, controllable rapid release, and the efficiency of direct solar energy utilization also affect the further development of azo‐based energy storage materials. Existing materials face challenges in balancing these factors. Achieving this balance through rational design to meet practical application needs is a key focus for future development. With ongoing research, it is expected that visible light‐responsive azo smart materials will increasingly contribute to advancements in the energy sector.

## CONFLICT OF INTEREST STATEMENT

The authors declare no conflicts of interest.

## Data Availability

Data sharing is not applicable to this article as no new data were created or analyzed in this study.
